# Atherothrombotic risk stratification after acute myocardial infarction: The Thrombolysis in Myocardial Infarction Risk Score for Secondary Prevention in the light of the French Registry of Acute ST Elevation or non‐ST Elevation Myocardial Infarction registries

**DOI:** 10.1002/clc.23131

**Published:** 2018-12-27

**Authors:** Etienne Puymirat, Marc Bonaca, Maxime Fumery, Victoria Tea, Nadia Aissaoui, Gilles Lemesles, Laurent Bonello, Grégory Ducrocq, Guillaume Cayla, Jean Ferrières, François Schiele, Tabassome Simon, Nicolas Danchin, for the FAST‐MI investigators

**Affiliations:** ^1^ Department of Cardiology Hôpital Européen Georges Pompidou (HEGP), Assistance Publique‐Hôpitaux de Paris (AP‐HP) Paris France; ^2^ Division of Cardiovascular Medicine TIMI Study Group, Brigham and Women's Hospital and Harvard Medical School Boston Massachusetts; ^3^ Department of Intensive Care AP‐HP, HEGP Paris France; ^4^ Department of Cardiology Lille Regional University Hospital Lille France; ^5^ Department of Cardiology Hôpital Nord, AP‐HM Marseille France; ^6^ Mediterranean Academic Association for Research and Studies in Cardiology (MARS Cardio), INSERM Aix‐Marseille University Marseille France; ^7^ Department of Cardiology, AP‐HP Hôpital Bichat Paris France; ^8^ Department of Cardiology CHU Nîmes Nîmes France; ^9^ Department of Cardiology Rangueil Hospital Toulouse France; ^10^ Department of Cardiology University Hospital Jean Minjoz Besançon France; ^11^ Department of Clinical Pharmacology and Unité de Recherche Clinique (URCEST) AP‐HP, Hôpital Saint Antoine, Université Pierre et Marie Curie (UPMC‐Paris 06) Paris France

**Keywords:** acute myocardial infarction, mortality, prevention, score

## Abstract

**Background:**

Guidelines recommend using risk stratification tools in acute myocardial infarction (AMI) to assist decision‐making. The Thrombolysis in Myocardial Infarction Risk Score for Secondary Prevention (TRS‐2P) has been recently developed to characterize long‐term risk in patients with MI.

**Hypothesis:**

We aimed to assess the TRS‐2P in the French Registry of Acute ST Elevation or non‐ST elevation MI registries.

**Methods:**

We used data from three 1‐month French registries, conducted 5 years apart, from 2005 to 2015, including 13 130 patients with AMI (52% ST‐elevation myocardial infarction [STEMI]). Atherothrombotic risk stratification was performed using the TRS‐2P score. Patients were divided in to three categories: G1 (low‐risk, TRS‐2P = 0/1); G2 (intermediate‐risk, TRS‐2P = 2); and G3 (high‐risk, TRS‐2P ≥ 3). Baseline characteristics and outcomes were analyzed according to TRS‐2P categories.

**Results:**

A total of 12 715 patients (in whom TRS‐2P was available) were included. Prevalence of G1, G2, and G3 was 43%, 24%, and 33% respectively. Clinical characteristics and management significantly differed according to TRS‐2P categories. TRS‐2P successfully defined residual risk of death at 1 year (C‐statistic 0.78): 1‐year survival was 98% in G1, 94% in G2, and 78.5% in G3 (*P* < 0.001). Using Cox multivariate analysis, G3 was independently associated with higher risk of death at 1 year (hazard ratio [HR] 4.61; 95% confidence interval [CI]: 3.61‐5.89), as G2 (HR 2.08; 95% CI: 1.62‐2.65) compared with G1. The score appeared robust and correlated well with mortality in STEMI and NSTEMI populations, as well as in each cohort separately.

**Conclusions:**

The TRS‐2P appears to be a robust risk score, identifying patients at high risk after AMI irrespective of the type of MI and historical period.

## 1 INTRODUCTION

Risk stratification tools enable personalized risk assessment and may help guide therapeutic decision‐making. Guidelines recommend their use in acute myocardial infarction (AMI) to identify high‐risk patients and to assist with short‐term prognostication and therapeutic decision‐making (eg, early invasive strategy).^1^, ^2^, ^3^, ^4^, ^5^, ^6^, ^7^, ^8^ Several scores have been developed, especially in patients at the acute stage of MI; however, they remain underutilized in clinical practice in part they require specific tools as well as a perception that the impact on treatment decisions is limited, or both. The Thrombolysis in Myocardial Infarction (TIMI) Risk Score for Secondary Prevention (TRS‐2P) is a simple nine‐point risk stratification tool, derived in patients with previous MI to predict recurrent cardiovascular (CV) events.^9^, ^10^, ^11^ This score has the advantage of being very simple to use and may assist with decisions on long‐term response to treatment. Recently, the TRS‐2P was validated in a clinical trial of acute coronary syndrome (ACS) patients followed for ~7 years.^12^ To our knowledge, the TRS‐2P score has never been evaluated in a routine‐practice population, focusing on patients who are discharged after an AMI. The aim of the present study was to test its robustness in several historical cohorts of patients after AMI, using the French Registry of Acute ST Elevation or non‐ST elevation Myocardial Infarction (FAST‐MI) registries.

## 2 METHODS

### 2.1 Patient population

Three nationwide French registries were conducted 5 years apart over a 10‐year period (2005‐2015): FAST‐MI 2005 (NCT00673036),^13^ FAST‐MI 2010 (NCT01237418),^14^ and FAST‐MI 2015 (NCT02566200)^15^ (Supporting Information Methods S1). The methods used for these registries have been detailed previously.^13^, ^14^, ^15^ Briefly, their primary objectives were to evaluate the characteristics, management, and outcomes of AMI patients, as seen in routine clinical practice, on a country‐wide scale.

All registries consecutively included patients with ST‐elevation myocardial infarction (STEMI) or non‐ST‐elevation myocardial infarction (NSTEMI) admitted to cardiac intensive care units (ICUs) within 48 hours of symptom onset, during a specified 1‐month period (October‐December 2005, 2010, and 2015). AMI was defined by increased levels of cardiac biomarkers (troponins, creatine kinase (CK), or creatine kinase‐MB (CK‐MB)) together with either compatible symptoms or electrocardiography (ECG) changes. Patients who died soon after admission and for whom cardiac markers were not measured were included if they had signs or symptoms associated with typical ST‐segment changes. Exclusion criteria were as follows: (a) refusal to participate, (b) iatrogenic MIs, defined as occurring within 48 hours of any therapeutic procedure, and (c) AMI diagnosis invalidated in favor of another diagnosis. STEMI was diagnosed when ST‐elevation ≥1 mm was seen in at least two contiguous leads in any location on the index or qualifying ECG, or when presumed new left bundle branch block or documented new Q waves were observed. In the absence of ST‐segment elevation, patients meeting the inclusion criteria were considered to have NSTEMI. A total of 13 130 patients (52% STEMI) were included in the three surveys.

Participation in the study was offered to all institutions, including university teaching hospitals, general and regional hospitals, and private clinics that received AMI emergencies. Physicians were instructed that the study should not affect clinical care or management. The study was conducted in accordance with the guidelines on good clinical practice and French law. The study protocols for the 2005 registry was reviewed by the CPP in Biomedical Research of Saint Antoine University Hospital; the 2010 registry was reviewed and approved by the CPP of Saint Louis University Hospital, Paris; and the protocol of 2015 registry was reviewed and approved by the CPP of Saint Louis University Hospital Paris Ile de France IV. Data file collection and storage were approved by the Commission Nationale Informatique et Liberté. All patients were informed of the nature and aims of the surveys and could request to be excluded; in addition, written consent was obtained for all three surveys.

### 2.2 Data collection

Data on baseline characteristics, including demographics (age, sex, body mass index), risk factors (hypertension, diabetes, current smoking, hypercholesterolemia, family history of coronary artery disease), and medical history (MI, previous myocardial revascularization, stroke, heart failure, peripheral artery disease [PAD], chronic renal failure), were collected as previously described.^13^, ^14^, ^15^ Information on the use of cardiac procedures, including use of percutaneous coronary intervention (PCI), use of medications (anticoagulants, antiplatelet agents, diuretics, beta‐blockers, angiotensin‐converting enzyme inhibitors (ACE‐I) or angiotensin receptor blockers (ARB), and lipid lowering agents) in the first 48 hours and at‐hospital discharge was collected.

Bleeding was classified as major or minor according to the TIMI criteria.^16^ Regarding bleeding complications, four end points of interest were used: in‐hospital major bleeding (defined as a fall in hemoglobin ≥5 g, fall in hematocrit ≥15%, intracranial hemorrhage, retroperitoneal bleeding), minor bleeding (defined as a fall in hemoglobin between 3 and 5 g/dL, fall in hematocrit between 10% and 15%), use of any transfusion during the hospital stay, and 1‐year survival.

For all surveys, follow‐up was centralized at the French Society of Cardiology.

### 2.3 Statistical analysis

Each patient was assessed for the presence of any of the nine previously described risk indicators in the Thrombin Receptor Antagonist in Secondary Prevention of Atherothrombotic Ischemic Events [TRA‐2P]—TIMI 50 trial at baseline^9^, ^10^, ^11^: age ≥ 75 years, diabetes mellitus, hypertension, PAD, previous stroke, previous coronary artery bypass grafting, history of heart failure, active smoking, and renal dysfunction (defined by an estimated glomerular filtration rate < 60 mL/min/1.73 m^2^ (using the Modification of Diet in Renal Disease equation). All variables, with the exception of age and renal dysfunction, were determined on the basis of clinical history. As described, each atherothrombotic risk indicator was weighted evenly to define total risk for each patient as the arithmetic sum of risk indicators. Simple risk categories were defined to parallel the annualized risk of death observed in the derivation population from patients in TRA2P, thus translating to a low‐risk category with 0 to 1 risk indicators (Group 1), an intermediate‐risk category with 2 risk indicators (Group 2), and a high‐risk category with ≥3 risk indicators (Group 3). The discriminatory capacity of the risk indicators was assessed by the area under the receiver operating characteristics curve (c‐statistic) as a measure of model performance.

Continuous variables are reported as means (SDs) or medians and interquartile ranges, when appropriate. Discrete variables are described as counts and percentages. Groups were compared by analysis of variance for continuous variables and *χ*2 (or Fisher exact tests) for discrete variables. Temporal trends were tested using linear‐by‐linear association tests for binary and Jonckheere‐Terpstra tests for continuous variables. Odds ratios and hazard ratios (HRs) are presented with their 95% confidence intervals (CIs).

Multivariable analyses of correlates of 1‐year mortality were performed using Cox backward stepwise multiple logistic regression, using a threshold of 0.10 for variable elimination. Beside time period, variables included in the final models were selected ad hoc, based on their physiological relevance and potential to be associated with outcomes; they comprised age, gender, risk factors, comorbidities, type of MI, TRS‐2P categories, year, and management. Sensitivity analyses were performed focused on patients discharged alive in the main analysis, inpatients with STEMI or NSTEMI separately, and in each of the three historical cohorts. Analyses were repeated using forward stepwise analysis to check the consistency of the results. Statistical analyses were performed using IBM SPSS 23.0 (IBM SPSS Inc., Chicago, IL). For all analyses, two‐sided *P* values <0.05 were considered significant.

## 3 RESULTS

### 3.1 Study population

A total of 12 715 patients (97%) had all nine variables included in the TRS‐2P score available at discharge and were included in the main analysis. Prevalence of Groups 1, 2, and 3 was 43%, 24%, and 33%, respectively. Over the 10‐year period, the overall risk of patients admitted for AMI decreased, with the proportion in Group 3 declining from 43% to 29% (*P* < 0.001; Figure S1). The distribution of the nine variables according to the TRS‐2P categories is presented in Figure S2. TRS‐2P successfully defined patients with high‐, intermediate‐, and low‐CV risk profile (Table 1). GRACE score was 168 ± 36 in Group 3, 139 ± 31 in Group 2, and 127 ± 27 in Group 1 (*P* < 0.001); simple risk index (SRI) was 35 ± 17, 26 ± 13, and 20 ± 10 in Groups 3, 2, and 1, respectively. In addition, the risk for major bleeding defined by the CRUSADE score decreased from Group 3 to Group 1.

**Table 1:** Baselines characteristics and clinical presentation

	Overall (*n* = 12 715)	Low (0–1) (*n* = 5446)	Intermediate (2) (*n* = 3108)	High (≥3) (*n* = 4161)	*P* value
Age (y)	65.9 ± 14.1	58.7 ± 11.8	66.0 ± 13.4	75.4 ± 11.5	<0.001
Female	3612 (28)	1110 (20)	916 (29.5)	1586 (38)	<0.001
BMI (kg/m^2^)	27.0 ± 4.7	26.6 ± 4.3	27.3 ± 4.9	27.4 ± 5.1	<0.001
Risk factors					
Hypertension	7016 (55)	1096 (20)	2252 (72.5)	3668 (88)	<0.001
Diabetes	3168 (25)	188 (3.5)	745 (24)	2235 (54)	<0.001
Hypercholesterolemia	5718 (45)	1939 (36)	1482 (48)	2297 (55)	<0.001
Current smoking	4234 (33)	1988 (36.5)	1229 (39.5)	1017 (24)	<0.001
Family history	3041 (24)	1671 (31)	754 (24)	616 (15)	<0.001
Medical history					
Prior MI	2214 (17)	498 (9)	505 (16)	1211 (29)	<0.001
Prior PCI	2041 (16)	513 (9)	491 (16)	1037 (25)	<0.001
Prior CABG	651 (5)	43 (0.8)	101 (3)	507 (12)	<0.001
History of heart failure	634 (5)	14 (0.3)	55 (2)	565 (14)	<0.001
History of stroke	785 (6)	56 (1)	132 (4)	597 (14)	<0.001
Peripheral artery disease	1064 (8)	28 (0.5)	127 (4)	909 (22)	<0.001
Chronic renal failure	638 (5)	23 (0.4)	65 (2)	550 (13)	<0.001
Prior medications					
Aspirin	3085 (24)	653 (12)	712 (23)	1720 (41)	<0.001
Clopidogrel	1394 (11)	214 (4)	272 (9)	908 (22)	<0.001
Beta‐blockers	3207 (25)	683 (12.5)	874 (28)	1650 (40)	<0.001
Statins	3648 (29)	954 (17.5)	924 (30)	1770 (42.5)	<0.001
ACE‐inhibitors or ARB	4305 (34)	848 (16)	1228 (39.5)	2229 (53)	<0.001
Clinical presentation					
STEMI	6650 (52)	3365 (62)	1670 (54)	1615 (39)	<0.001
Killip class					<0.001
I	10 544 (83)	5331 (98)	2770 (89)	2443 (59)	
II	1227 (10)	81 (1.5)	224 (7)	922 (22)	
III	714 (6)	14 (0.3)	71 (2)	629 (15)	
IV	180 (1)	10 (0.2)	28 (0.9)	142 (3.4)	
LV function	51.7 ± 11.7	53.8 ± 10.3	52.4 ± 11.4	48.3 ± 12.9	<0.001
GRACE score	143.3 ± 36.0	126.5 ± 26.7	139.2 ± 31.1	167.9 ± 36.2	<0.001
SRI score					<0.001
Median (IQR)		18.3 (13.3‐24.7)	22.8 (16.3‐31.7)	32.6 (24.0‐43.0)	
*n*		5283	3025	4065	
CRUSADE					<0.001
Median (IQR)		18.0 (9.0‐26.0)	26.0 (16.0‐36.0)	44.1 (33.0‐53.0)	
*n*		5041	2868	3764	
CRP (mg/L)					<0.001
Median (IQR)		4.0 (2.0‐8.9)	5.0 (2.9‐11.4)	8.8 (4.0‐33.0)	
*n*		4025	2307	3153	

Abbreviations: ACE, angiotensin‐converting enzyme; ARB, angiotensin receptor blockers; BMI, body mass index; CABG, coronary artery bypass grafting; CRP, C‐reactive protein; LV, left ventricular; MI, myocardial infarction; NSTEMI, Non‐ST‐elevation myocardial infarction; PCI, percutaneous coronary intervention; SRI, simple risk index; STEMI, ST‐elevation myocardial infarction.

Values are expressed as mean (± SD) or number (percentage).

The rate of STEMI patients was higher in Group 1, while the rate of patients with heart failure at admission (Killip class ≥ 2) was higher in Group 3. Interestingly, biomarkers of inflammation (eg, C‐reactive protein) increased from Group 1 to Group 3.

### 3.2 Early management

Early management including medications and myocardial revascularization were significantly different according to TRS‐2P categories (Table 2). Overall, Group 3 patients received less antiplatelet agents, statin, beta‐blocker, ACE‐I, or ARB during the first 48 hours after admission as at discharge compared with both Groups 1 and 2 (*P* < 0.001 for all). In Group 3 patients, the use of appropriate secondary prevention treatment (dual antiplatelet therapy and statins for all; ACE‐I/ARB and beta‐blockers as indicated) was lower especially in patients with renal dysfunction (42% vs 55%, *P* < 0.001) and older patients (<60 years: 60%; 60‐74 years: 53%; ≥75 years: 45%; *P* = 0.001). In addition, the use of invasive strategy (coronary angiography with or without PCI) was lower in Group 3, in which the rate of multivessel disease was higher. Radial access was preferentially used in low‐ or intermediate‐risk patients. Finally, a full myocardial revascularization strategy during hospitalization was more frequently used in both Groups 1 and 2 (*P* < 0.001).

**Table 2:** In‐hospital management

	Overall (*n* = 12 715)	Low (0‐1) (*n* = 5446)	Intermediate (2) (*n* = 3108)	High (≥3) (*n* = 4161)	*P* value
Medications					
Aspirin	11 767 (92.5)	5126 (94)	2905 (93.5)	3736 (90)	<0.001
Clopidogrel	7627 (60)	2891 (53)	1909 (61)	2827 (68)	<0.001
Ticagrelor	2483 (19.5)	1365 (25)	613 (20)	505 (12)	<0.001
Prasugrel	1683 (13)	1113 (20)	384 (12)	186 (4.5)	<0.001
GPIIb/IIIa	198 (2)	112 (2)	49 (2)	37 (0.9)	<0.001
UFH	5266 (41)	2098 (38.5)	1226 (39)	1942 (47)	<0.001
LMWH	6844 (54)	3223 (59)	1741 (56)	1880 (45)	<0.001
Bivalirudine	275 (2)	152 (3)	74 (2)	49 (1)	<0.001
Fondaparinux	1934 (15)	879 (16)	466 (15)	589 (14)	0.03
Statins	9820 (77)	4486 (82)	2429 (78)	2905 (70)	<0.001
Beta‐blockers	9390 (74)	4305 (79)	2361 (76)	2724 (65.5)	<0.001
ACE‐inhibitors or ARB	7610 (60)	3195 (59)	1958 (63)	2457 (59)	<0.001
Procedures					
CAG	11 800 (93)	5381 (99)	2975 (96)	3444 (83)	<0.001
CAG results					<0.001
No significant lesions (<50%)	718 (6)	384 (7)	175 (6)	159 (4)	
One‐VD	4830 (38)	2642 (49)	1212 (39)	976 (24)	
Two‐VD	3436 (27)	1522 (28)	898 (29)	1016 (24)	
Three‐VD	2156 (17)	755 (14)	575 (18.5)	826 (20)	
CABG	603 (5)	42 (1)	99 (3)	462 (11)	
PCI	7819 (66)	3976 (74)	2009 (68)	1834 (53)	<0.001
Radial access	7134 (78)	3577 (84)	1795 (80)	1762 (67)	<0.001
Drug‐eluting stent	5084 (48)	2494 (51)	1293 (48.5)	1297 (43)	<0.001
Complete myocardial revascularization	5588 (61)	2879 (66)	1428 (61)	1273 (52)	<0.001

Abbreviations: ACE, angiotensin‐converting enzyme; ARB, angiotensin receptor blockers; CABG, coronary artery bypass grafting; CAG, coronary angiography; UFH, unfractionned heparin; LMWH, low‐molecular‐weight heparin; NSTEMI, non‐ST‐elevation myocardial infarction; PCI, percutaneous coronary intervention; STEMI, ST‐elevation myocardial infarction; TIMI, thrombolysis in myocardial infarction; VD, vessel disease.

Values are expressed as mean (± SD) or number (percentage).

### 3.3 Outcomes

In‐hospital complications are described in (Table 3). The rate of re‐MI, atrial fibrillation, stroke, and major and minor bleedings were higher in Group 3 patients. Mortality at 30 days was 9% in Group 3, 3% in Group 2, and 1% in Group 1 (*P* < 0.001).

**Table 3:** In‐hospital complications and clinical outcomes

	Overall (*n* = 12 715)	Low (0‐1) (*n* = 5446)	Intermediate (2) (*n* = 3108)	High (≥3) (*n* = 4161)	*P* value
Re‐MI	130 (1.0)	32 (0.6)	26 (0.8)	72 (2)	<0.001
Intrastent thrombosis	53 (0.6)	25 (0.6)	12 (0.5)	16 (0.6)	<0.001
Atrial fibrillation	770 (6)	174 (3)	167 (5)	429 (10)	<0.001
Ventricular fibrillation	245 (2)	110 (2)	54 (2)	81 (2)	0.66
Stroke	75 (0.6)	16 (0.3)	16 (0.5)	43 (1.0)	<0.001
Major bleeding	259 (2)	77 (1)	53 (2)	129 (3)	<0.001
Minor bleeding	360 (3)	124 (2)	90 (3)	146 (3.5)	0.001
Transfusions	436 (3)	69 (1)	87 (3)	280 (7)	<0.001
Death at 30 days	502 (3.9)	49 (0.9)	83 (2.7)	370 (8.9)	<0.001
Death at 1 year	1197 (9)	127 (2.3)	177 (5.7)	893 (21.5)	<0.001

Abbreviations: MI, myocardial infarction; NSTEMI, non‐ST‐elevation myocardial infarction; STEMI, ST‐elevation myocardial infarction.

Values are expressed as mean (± SD) or number (percentage).

**Figure 1:**
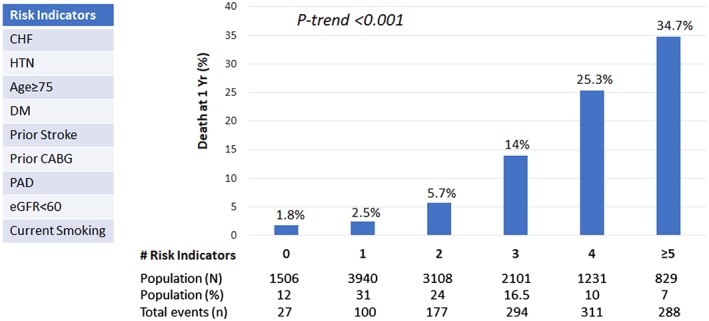
Risk stratification of death at 1 year. One‐year Kaplan‐Meier estimates are shown. The *P* value is based on the *χ* ^2^ test for trend. CABG, coronary artery bypass graft; CHF, congestive heart failure; DM, diabetes mellitus; eGFR, estimated glomerular filtration rate; HTN, hypertension; PAD, peripherical artery disease

**Figure 2:**
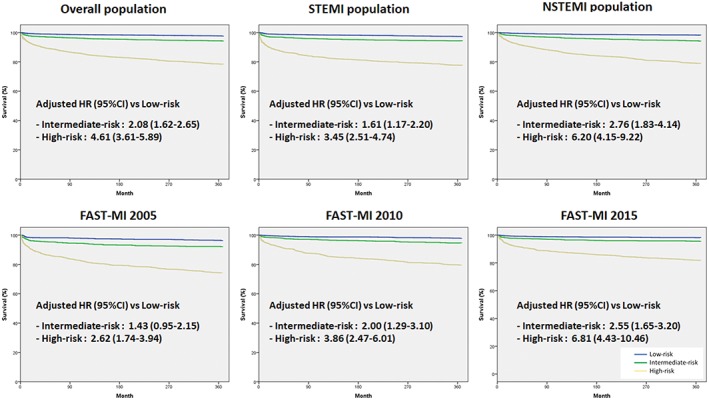
One‐year mortality according to Thrombolysis in Myocardial Infarction Risk Score for Secondary Prevention (TRS2P) categories. The survival curves are unadjusted, and the adjusted hazard ratios (HRs) are provided with their 95% confidence intervals (CIs). NSTEMI, non‐ST‐elevation myocardial infarction; STEMI, ST‐elevation myocardial infarction

Distribution of patients across the full range of risk indicators is provided in (Figure 1). TRS 2P score successfully defined residual risk of death at 1 year (C‐statistic 0.78): 1‐year survival was 98% in Group 1, 94% in Group 2, and 78.5% in Group 3 (*P* < 0.001). Using Cox multivariate analysis, Group 3 and Group 2 were associated with a higher risk of death at 1‐year (HR = 4.61; 95% CI: 3.61‐5.89, *P* < 0.001, and HR = 2.08; 95% CI: 1.62‐2.65, *P* < 0.001, respectively) compared with Group 1 (Figure 2). Similar trends were found after censoring patients dying during hospitalization.

### 3.4 Subgroup analyses

Similar trends were found using TRS‐2P score according to type of MI and year of survey (Tables S1‐S6; Figure S2). The score appeared robust and correlated well with mortality in STEMI (C‐statistic 0.77) and NSTEMI (c‐statistic 0.78) populations, as well as in each of the historical cohorts separately: 2005 (c‐statistic 0.76), 2010 (c‐statistic 0.78) and 2015 (c‐statistic 0.78).

## 4 DISCUSSION

The main findings of this study are that the easily calculated TRS‐2P appears to be a robust risk score, identifying patients at high‐risk after AMI, irrespective of the type of MI and historical period. In addition, we showed that the rate of high‐risk patients among those hospitalized for AMI decreased over the 10‐year period from 2005 to 2015.

### 4.1 Change in risk‐profile

Several sources including registries specific to AMI and large databases, have shown a decrease in mortality over the past 20 years.^17^, ^18^, ^19^, ^20^, ^21^ Using the FAST MI program, we previously reported that this decrease was correlated partially with a substantial change in patient risk profile, and not only with changes in management. Specifically, the absolute 6‐month mortality decrease from 1995 to 2015 was 11.9% (observed) vs 10.1% (standardized), attesting a 15% reduction related to the changes in patient risk profile in STEMI patients (17% in NSTEMI patients).^21^ In the present analysis, the rate of high‐risk patients in TRS2P score decreased by 32% over the 10‐year period.

### 4.2 Atherothrombotic risk assessment

Patients after AMI demonstrate a range of residual risk for recurrent CV events. Therefore, risk stratification tools have been developed, usually derived from clinical trials or specific registries to identify high‐risk patients and to assist with prognostication and therapeutic decision making. A limited number of risk scores, however, are available for patients in secondary prevention after AMI. The TRS‐2P score has been proposed by the TIMI group for stable ischemic heart disease (IHD) using data from the TRA2P‐TIMI 50 trial.^9^, ^10^, ^11^ Nine independent risk predictors were identified in this cohort. These variables are highly consistent with those used for other risk scores. Diabetes mellitus, hypertension, and current smoking are established risk factors for disease progression recognized by stable IHD practice guidelines as high‐risk comorbid conditions warranting particular focus for medical therapy. Moreover, the presence of atherosclerosis outside the coronary bed, heart failure and renal dysfunction are also now well recognized as potent risk indicators across the spectrum of IHD. The trial showed a strong graded relationship with the rate of CV death/MI/ischemic stroke and the individual components. The TRS‐2P score was, however, defined in a selected population in which for example, women and minorities made up a small proportion of the study population.^9^, ^10^, ^11^ Recently, the TRS‐2P score has been validated in routine practice using two large, independent integrated healthcare delivery systems in United States between 2008 and 2013 (ie, Cleveland Clinic and Geisinger Health System).^22^ However, to our knowledge, this stratification tool has never been evaluated in a routine‐practice population, focusing on patients who are discharged alive after an AMI. In our analysis, the TRS‐2P score appears to be a robust risk score to identify high risk patients irrespective of the type of MI and historical period. Moreover, this score appears relevant in patients hospitalized for an acute MI (eg, not only in stable CAD or patients having sustained an infarct in the previous year).

The TRS‐2P has never been compared with Global Registry of Acute Coronary Events (GRACE), SRI or Can Rapid risk stratification of Unstable angina patients Suppress Adverse outcomes with Early implementation of the ACC/AHA Guidelines (CRUSADE) scores. Our data show that TRS‐2P score is consistent with these validated scores to define a high‐risk population (ie, Group 3) in terms of bleeding risk and ischemic risk.

### 4.3 Risk assessment and therapeutic intensification

Atherothrombotic risk assessment may be useful to identify high‐risk patients who have the greatest potential to benefit from more intensive secondary prevention therapy such as antithrombotic or lipid‐lowering. In the TRA2P‐TIMI 50 trial, the risk stratification tool identified a gradient of risk for recurrent events and distinguished a pattern of increasing absolute benefit with vorapaxar.^9^, ^10^, ^11^ Similarly, using data from the IMPROVE IT trial, the TRS‐2P score identified a strong gradient of risk for recurrent CV events; and an increasingly favorable relative and absolute benefit from the addition of ezetimibe to simvastatin therapy with increasing risk‐profile.^12^ Finally, this score could be evaluated to identify high‐risk patients for new strategies as PCSK9‐inhibitors or prolonged double antiplatelet therapy in ACS patients.^23^, ^24^ Yet, in clinical practice, the highest risk patients are paradoxically often the least intensively treated.

### 4.4 Limitations

The TRS‐2P score was designed to be a simple tool, using readily available clinical data. There are other previously identified risk indicators and other yet to be identified parameters that may provide additional refinement for stratification. However, the ability of this simple scoring system to identify differential treatment benefit for different classes of secondary prevent therapy supports its clinically utility. Our data are derived from an observational study of AMI patients admitted in ICUs while TRS‐2P was defined in a population of stable patients with previous MI. In addition, our analyses were focused on the mortality at one‐year while this risk stratification tool was developed for all CV‐events at 3‐year. The rate of CV death was not available. Finally, we cannot exclude that other factor than those collected in the surveys could also explain the evolution observed according to TRS2P categories.

## 5 CONCLUSION

Atherothrombotic risk assessment may be useful to identify high‐risk patients who have the greatest potential to benefit from more intensive secondary preventive therapy. Using a routine‐practice population, TRS‐2P appears to be a robust risk score, identifying patients at high‐risk after AMI irrespective of the type of MI and historical period.

## Supporting information

Appendix S1.
