# Supplementary material for: Atherothrombotic risk stratification after acute myocardial infarction: The Thrombolysis in Myocardial Infarction Risk Score for Secondary Prevention in the light of the French Registry of Acute ST Elevation or non‐ST Elevation Myocardial Infarction registries
Source: Clin Cardiol. 2018 Dec 27;42(2):227–34. doi: 10.1002/clc.23131 (PMC6712320; doi:10.1002/clc.23131)
Supplement: Supplementary file 1 — Appendix S1. [file CLC-42-227-s001.docx]

**Online FILES**

**Online Methods 1.** Methodology of the 3 surveys.

**Online Table 1.** Baseline characteristics according to acute myocardial infarction

**Online Table 2.** In-hospital management according to acute myocardial infarction

**Online Table 3.** In-hospital complications and clinical outcomes according to acute myocardial infarction

**Online Table 4.** Baseline characteristics according to survey

**Online Table 5.** In-hospital management according to survey

**Online Table 6.** In-hospital complications and clinical outcomes according to survey

**Online Figure 1.** TRS-2P categories between 2005 to 2015

**Online Figure 2.** Distribution of the 9 variables according to TRS-2P categories

**Online Methods 1.** Methodology of the 3 surveys.

**FAST-MI 2005 (NCT00673036):**

- Sponsor: French Society of Cardiology
- Principal investigators: N. Danchin, T. Simon
- Funding: Pfizer, Servier, and additional grant from the French National Health Insurance (CNAM-TS).
- The protocol was reviewed by the Committee for the Protection of Human Subjects in Biomedical Research of Saint Antoine University Hospital (Paris, France).
- Aim: to evaluate practices for AMI management in "real life" practice, and to measure their impact on the medium- and long-term outcomes of patients admitted to intensive care units for AMI over a one-month period in France.
- Institutions: voluntary participation of any institution authorised to take care of AMI patients, i.e. university hospitals, general hospitals, private clinics, with or without catheterization laboratory.
- Patient population: consecutive adult patients admitted to the participating centres and meeting the following criteria:
  - Inclusion criteria:
    - Diagnosis of AMI on the basis of elevated CK-MB or troponin, in combination with:
      - Symptoms compatible with prolonged myocardial ischaemia
      - Or ECG changes compatible with myocardial ischaemia: pathologic new Q waves or ST elevation, ST depression, or T wave inversion
    - Time from onset to admission < 48 hours
    - Patients who died very early after admission and for whom cardiac markers were not measured or not yet elevated were included if they had compatible signs or symptoms associated with typical ST changes.
  - Exclusion criteria:
    - Iatrogenic myocardial infarction
    - AMI diagnosis invalidated in favour of another diagnosis
- Period of inclusion: study start in the participating centres between October 1^st^ and November 15^th^ 2005, inclusion for 31 consecutive days. For diabetic patients, inclusion was prolonged for one additional month.
- Electronic case record form with automated data queries, filled-in by dedicated research technicians sent at each site at least once a week. Details (exact type and dose) of all medications prescribed at different time-points. Blood collection (DNA and serum) for core laboratory analysis, in the largest centres.

Ten-year follow-up centralised at the French Society of Cardiology and carried-out by dedicated research technicians (URCEST, APHP, Paris, France). All reported events reviewed by a critical events committee (Nicolas Danchin, Pascal Guéret, Tabassome Simon). The database is cross-linked with the French Institute for National Statistics and the French national death database (CepiDC) to gather additional information on the cause of death.

**FAST-MI 2010 (NCT01237418):**

- Sponsor: French Society of Cardiology
- Principal investigators: N. Danchin, T. Simon
- Funding: AstraZeneca, the Daiichi-Sankyo/Eli-Lilly alliance, GSK, MSD, Novartis, Sanofi.
- The protocol was reviewed and approved by the Committee for the Protection of Human Subjects of Saint Louis University Hospital (Paris, France).
- Aim: to provide an extensive description of the population of patients admitted for AMI throughout the French territory, to determine whether differences in terms of population characteristics existed across regions, to assess the management of the patients suffering from AMI, and to determine the implementation of practice guidelines in a real world setting. Other objectives were to assess the correlations between management strategies and outcomes, to determine the correlations between genetic polymorphisms and morbi-mortality in relation with the effects of medications, and to determine relationships between biomarkers and morbi-mortality. Another objective was to enable historic comparisons with the previous French registries.
- Institutions: voluntary participation of any institution authorised to take care of AMI patients, i.e. university hospitals, general hospitals, private clinics, with or without catheterization laboratory.
- Patient population: consecutive adult patients admitted to the participating centres and meeting the following criteria:
  - Inclusion criteria:
    - Diagnosis of acute myocardial infarction on the basis of elevated CK-MB or troponin, in combination with:
      - Symptoms compatible with prolonged myocardial ischaemia
      - Or ECG changes compatible with myocardial ischaemia: pathologic new Q waves or ST elevation, ST depression, or T wave inversion
    - Time from onset to admission < 48 hours
    - Patients who died very early after admission and for whom cardiac markers were not measured or not yet elevated were included if they had compatible signs or symptoms associated with typical ST changes.
  - Exclusion criteria:
    - Iatrogenic myocardial infarction
    - AMI diagnosis invalidated in favour of another diagnosis
- Period of inclusion: study start in the participating centres between October 1^st^ and November 30^th^ 10, inclusion for 31 consecutive days. Centres which were willing to do so could prolong recruitment up to one additional month.
- Electronic case record form with automated data queries, filled-in by dedicated research technicians sent at each site at least once a week. Details (exact type and dose) of all medications prescribed at different time-points. Blood collection (DNA and serum) for core laboratory analysis, in the largest centres.

Ten-year follow-up centralised at the French Society of Cardiology, and done by dedicated research technicians (URCEST, APHP, Paris, France). All reported events reviewed by a critical events committee (Nicolas Danchin, Pascal Guéret, Tabassome Simon). The database is cross-linked with the French Institute for National Statistics and the French national death database (CepiDC) to gather additional information on the cause of death.

**FAST-MI 2015 (NCT02566200):**

- Sponsor: French Society of Cardiology
- Principal investigators: N. Danchin, T. Simon
- Funding: Amgen, AstraZeneca, Bayer, BMS, Boehringer Ingelheim, the Daiichi-Sankyo/Eli-Lilly alliance, MSD, Pfizer, Sanofi.
- The protocol was reviewed and approved by the Committee for the Protection of Human Subjects of Saint Louis University Hospital (Paris, France).
- Aim: to provide an extensive description of the population of patients admitted for AMI throughout the French territory, to determine whether differences in terms of population characteristics existed across regions, to assess the management of the patients suffering from AMI, and to determine the implementation of practice guidelines in a real world setting. Other objectives were to assess the correlations between management strategies and outcomes, to determine the correlations between genetic polymorphisms and morbi-mortality in relation with the effects of medications, and to determine relationships between biomarkers and morbi-mortality. Another objective was to enable historic comparisons with the previous French registries.
- Institutions: voluntary participation of any institution authorised to take care of AMI patients, i.e. university hospitals, general hospitals, private clinics, with or without catheterization laboratory.
- Patient population: consecutive adult patients admitted to the participating centres and meeting the following criteria:
  - Inclusion criteria:
    - Diagnosis of acute myocardial infarction on the basis of elevated CK-MB or troponin, in combination with:
      - Symptoms compatible with prolonged myocardial ischaemia
      - Or ECG changes compatible with myocardial ischaemia: pathologic new Q waves or ST elevation, ST depression, or T wave inversion
    - Time from onset to admission < 48 hours
    - Patients who died very early after admission and for whom cardiac markers were not measured or not yet elevated were included if they had compatible signs or symptoms associated with typical ST changes.
  - Exclusion criteria:
    - Iatrogenic myocardial infarction
    - AMI diagnosis invalidated in favour of another diagnosis
- Period of inclusion: study start in the participating centres between October 1^st^ and December 6^th^ 2015, inclusion for 31 consecutive days. Centres which were willing to do so could prolong recruitment up to one additional month.
- Electronic case record form with automated data queries, filled-in by dedicated research technicians sent at each site at least once a week. Details (exact type and dose) of all medications prescribed at different time-points. Blood collection (DNA and serum) for core laboratory analysis, in the largest centres.

Ten-year follow-up centralised at the French Society of Cardiology, and done by dedicated research technicians (URCEST, APHP, Paris, France). All reported events reviewed by a critical events committee (Nicolas Danchin, Pascal Guéret, Tabassome Simon). The database is cross-linked with the French Institute for National Statistics and the French national death database (CepiDC) to gather additional information on the cause of death.

**Contract Research Organisations:**

- **Clinical data:** 2005 and 2010: ICTA Dijon, France; 2015: Axonal, Nanterre, France

**Biology:** 2005, 2010 and 2015: CEMO, Choisy le Roi, France

**Data file collection and storage were approved by the Commission Nationale Informatique et Liberté for all registries.**

**Online Table 1.** Baseline characteristics according to acute myocardial infarction

|  | **Low (0-1)** | | | **Intermediate (2)** | | | **High (≥3)** | | | **P-value** | |
| --- | --- | --- | --- | --- | --- | --- | --- | --- | --- | --- | --- |
|  | **STEMI**  **(n=3365)** | **NSTEMI**  **(n=2081)** |  | **STEMI**  **(n=1670)** | **NSTEMI**  **(n=1438)** |  | **STEMI**  **(n=1615)** | **NSTEMI**  **(n=2546)** |  | **STEMI** | **NSTEMI** |
| Age (y) | 57.7±11.8 | 60.4±11.6 |  | 64.3±13.6 | 68.0±12.9 |  | 74.5±12.3 | 75.9±10.9 |  | <0.001 | <0.001 |
| Female | 619 (18) | 491 (24) |  | 477 (29) | 439 (31) |  | 633 (39) | 953 (37) |  | <0.001 | <0.001 |
| BMI (Kg/m²) | 26.4±4.2 | 26.8±4.3 |  | 27.3±4.8 | 27.2±4.9 |  | 27.2±5.0 | 27.5±5.2 |  | <0.001 | <0.001 |
| **Risk factors** |  |  |  |  |  |  |  |  |  |  |  |
| Hypertension | 568 (17) | 528 (25) |  | 1173 (75) | 1079 (70) |  | 1401 (87) | 2267 (89) |  | <0.001 | <0.001 |
| Diabetes | 99 (3) | 89 (4) |  | 381 (23) | 364 (25) |  | 815 (51) | 1420 (56) |  | <0.001 | <0.001 |
| Hypercholesterolemia | 1110 (33) | 829 (40) |  | 749 (45) | 733 (51) |  | 803 (50) | 1494 (59) |  | <0.001 | <0.001 |
| Current smoking | 1398 (42) | 590 (28) |  | 794 (48) | 435 (30) |  | 487 (30) | 530 (21) |  | <0.001 | <0.001 |
| Family History | 1021 (30) | 650 (31) |  | 425 (25) | 329 (23) |  | 230 (14) | 386 (15) |  | <0.001 | <0.001 |
| **Medical history** |  |  |  |  |  |  |  |  |  |  |  |
| Prior MI | 234 (7) | 264 (13) |  | 203 (12) | 302 (21) |  | 325 (20) | 886 (35) |  | <0.001 | <0.001 |
| Prior PCI | 237 (7) | 276 (13) |  | 185 (11) | 306 (21) |  | 275 (17) | 762 (30) |  | <0.001 | <0.001 |
| Prior CABG | 25 (0.7) | 18 (0.9) |  | 46 (3) | 55 (4) |  | 114 (7) | 393 (15) |  | <0.001 | <0.001 |
| History of heart failure | 5 (0.1) | 9 (0.4) |  | 18 (1) | 37 (3) |  | 157 (10) | 408 (16) |  | <0.001 | <0.001 |
| History of stroke | 27 (0.8) | 29 (1) |  | 69 (4) | 63 (4) |  | 218 (13.5) | 379 (15) |  | <0.001 | <0.001 |
| Peripheral artery disease | 15 (0.4) | 13 (0.6) |  | 57 (3) | 70 (5) |  | 264 (16) | 645 (25) |  | <0.001 | <0.001 |
| Chronic renal failure | 9 (0.4) | 9 (0.4) |  | 30 (2) | 35 (2) |  | 149 (9) | 401 (16) |  | <0.001 | <0.001 |
| **Prior medications** |  |  |  |  |  |  |  |  |  |  |  |
| Aspirin | 290 (9) | 363 (17) |  | 285 (17) | 427 (30) |  | 514 (32) | 1206 (47) |  | <0.001 | <0.001 |
| Clopidogrel | 95 (3) | 119 (6) |  | 88 (5) | 184 (13) |  | 225 (14) | 683 (27) |  | <0.001 | <0.001 |
| Betablockers | 308 (9) | 375 (18) |  | 399 (24) | 475 (33) |  | 522 (32) | 1128 (44) |  | <0.001 | <0.001 |
| Statins | 494 (15) | 460 (22) |  | 426 (26) | 498 (35) |  | 538 (33) | 1232 (48) |  | <0.001 | <0.001 |
| ACE-Inhibitors or ARB | 450 (13) | 398 (19) |  | 581 (35) | 647 (45) |  | 773 (48) | 1456 (57) |  | <0.001 | <0.001 |
| **Clinical presentation** |  |  |  |  |  |  |  |  |  |  |  |
| Killips class  I  II  III  IV | 3294 (98)  51 (1.5)  7 (0.2)  7 (0.2) | 2037 (98)  30 (1)  7 (0.3)  3 (0.1) |  | 1473 (88)  143 (9)  32 (2)  16 (1) | 1297 (90)  81 (6)  39 (3)  12 (0.8) |  | 975 (60)  378 (23)  168 (10)  87 (5) | 1468 (58)  544 (21)  461 (18)  55 (2) |  | <0.001 | <0.001 |
| LV function (%) | 52.0±10.1 | 56.7±9.9 |  | 50.8±11.1 | 54.2±11.5 |  | 46.9±12.5 | 49.3±13.0 |  | <0.001 | <0.001 |
| GRACE score | 131.4±24.4 | 118.5±28.4 |  | 144.3±29.5 | 133.2±31.9 |  | 175.2±35.4 | 163.3±36.0 |  | <0.001 | <0.001 |
| SRI score | 19.8±9.7 | 20.6±10.4 |  | 24.8±13.5 | 26.3±13.3 |  | 35.0±17.0 | 35.4±16.6 |  | <0.001 | <0.001 |
| CRUSADE | 18.6±10.9 | 19.3±11.4 |  | 25.4±13.0 | 26.9±12.9 |  | 41.4±14.3 | 43.6±15.1 |  | <0.001 | <0.001 |
| CRP | 13.9±37.4 | 10.5±25.1 |  | 17.3±37.8 | 14.9±34.3 |  | 33.6±54.1 | 32.4±55.9 |  | <0.001 | <0.001 |

Values are expressed as mean (± SD) or number (percentage)

ACE, angiotensin-converting enzyme; ARB, angiotensin receptor blockers; BMI, body mass index; CABG, coronary artery bypass grafting; CRP, C-reactive protein, LV, left ventricular; MI, myocardial infarction; NSTEMI, Non-ST-elevation myocardial infarction; PCI, percutaneous coronary intervention; SRI, simple risk index; STEMI, ST-elevation myocardial infarction.

**Online Table 2.** In-hospital management according to acute myocardial infarction

|  | **Low (0-1)** | | | **Intermediate (2)** | | | **High (≥3)** | | | **P-value** | |
| --- | --- | --- | --- | --- | --- | --- | --- | --- | --- | --- | --- |
|  | **STEMI**  **(n=3365)** | **NSTEMI**  **(n=2081)** |  | **STEMI**  **(n=1670)** | **NSTEMI**  **(n=1438)** |  | **STEMI**  **(n=1615)** | **NSTEMI**  **(n=2546)** |  | **STEMI** | **NSTEMI** |
| **Medications** |  |  |  |  |  |  |  |  |  |  |  |
| Aspirin | 3196 (95) | 1930 (93) |  | 1554 (93) | 1351 (94) |  | 1464 (91) | 2272 (89) |  | <0.001 | <0.001 |
| Clopidogrel | 1729 (51) | 1162 (56) |  | 1011 (60.5) | 898 (62) |  | 1100 (68) | 1727 (68) |  | <0.001 | <0.001 |
| Ticagrelor | 770 (23) | 595 (29) |  | 314 (19) | 299 (21) |  | 210 (13) | 295 (12) |  | 0.4 | 0.4 |
| Prasugrel | 918 (27) | 195 (9) |  | 294 (18) | 90 (6) |  | 122 (8) | 64 (2.5) |  | <0.001 | <0.001 |
| GPIIbIIIa | 100 (3) | 12 (0.6) |  | 43 (3) | 6 (0.4) |  | 27 (2) | 10 (0.4) |  | <0.001 | <0.001 |
| UFH | 1403 (42) | 695 (33) |  | 748 (45) | 478 (33) |  | 823 (51) | 1119 (44) |  | <0.001 | 0.06 |
| LMWH | 2009 (60) | 1214 (58) |  | 915 (55) | 826 (57) |  | 762 (47) | 1118 (44) |  | 0.006 | <0.001 |
| Bivalirudine | 134 (4) | 18 (0.9) |  | 60 (4) | 14 (1) |  | 38 (2) | 11 (0.4) |  | 0.09 | 0.01 |
| Fondaparinux | 422 (12.5) | 457 (22) |  | 193 (12) | 273 (19) |  | 184 (11) | 405 (16) |  | 0.41 | <0.001 |
| Statins | 2846 (85) | 1640 (79) |  | 1340 (80) | 1089 (76) |  | 1161 (72) | 1744 (68.5) |  | <0.001 | <0.001 |
| Beta-blockers | 2729 (81) | 1576 (76) |  | 1304 (78) | 1057 (73.5) |  | 1055 (65) | 1669 (66) |  | <0.001 | <0.001 |
| ACE-inhibitors or ARB | 2125 (63) | 1070 (51) |  | 1077 (64.5) | 881 (61) |  | 940 (58) | 1517 (60) |  | <0.001 | <0.001 |
| **Procedures** |  |  |  |  |  |  |  |  |  |  |  |
| CAG | 3340 (99) | 2041 (98) |  | 1616 (97) | 1359 (94.5) |  | 1408 (87) | 2036 (80) |  | <0.001 | <0.001 |
| PCI | 3011 (89.5) | 1473 (71) |  | 1485 (88) | 963 (67) |  | 1195 (74) | 1309 (51) |  | <0.001 | <0.001 |
| Reperfusion therapy in STEMI patients  Primary PCI  Fibrinolysis  Medical therapy | 2071 (61.5)  568 (17)  726 (22) | - |  | 997 (60)  244 (15)  428 (26) | - |  | 795 (49)  165 (10)  655 (41) | - |  | <0.001 | - |
| CAG < 24hours in NSTEMI patients | - | 1078 (53) |  | - | 640 (47) |  | - | 740 (36) |  | - | <0.001 |

Values are expressed as mean (± SD) or number (percentage)

ACE, angiotensin-converting enzyme; ARB, angiotensin receptor blockers; CAG, coronary angiography; LMWH, low-molecular-weight heparin;

LV, left ventricular; MI, myocardial infarction; NSTEMI, Non- ST-elevation myocardial infarction; PCI, percutaneous coronary intervention; STEMI, ST-elevation myocardial infarction; UFH, unfractionated heparin.

**Online Table 3.** In-hospital complications and clinical outcomes according to acute myocardial infarction

|  | **Low (0-1)** | | | **Intermediate (2)** | | | **High (≥3)** | | | **P-value** | |
| --- | --- | --- | --- | --- | --- | --- | --- | --- | --- | --- | --- |
|  | **STEMI**  **(n=3365)** | **NSTEMI**  **(n=2081)** |  | **STEMI**  **(n=1670)** | **NSTEMI**  **(n=1438)** |  | **STEMI**  **(n=1615)** | **NSTEMI**  **(n=2546)** |  | **STEMI** | **NSTEMI** |
| Re-MI | 27 (0.8) | 5 (0.2) |  | 11 (0.7) | 15 (1) |  | 23 (1) | 49 (2) |  | 0.04 | <0.001 |
| Intrastent thrombosis | 22 (0.8) | 3 (0.2) |  | 6 (0.5) | 6 (0.6) |  | 10 (1) | 6 (0.4) |  | 0.41 | 0.24 |
| Atrial fibrillation | 122 (4) | 52 (2.5) |  | 100 (6) | 67 (5) |  | 192 (12) | 237 (9) |  | <0.001 | <0.001 |
| Ventricular fibrillation | 91 (3) | 19 (0.9) |  | 48 (3) | 6 (0.4) |  | 51 (3) | 30 (1) |  | 0.05 | 0.67 |
| Stroke | 13 (0.4) | 3 (0.1) |  | 12 (0.7) | 4 (0.3) |  | 20 (1.2) | 23 (0.9) |  | 0.003 | <0.001 |
| Major bleeding | 44 (1) | 33 (2) |  | 31 (2) | 22 (1.5) |  | 59 (4) | 70 (3) |  | <0.001 | <0.001 |
| Minor bleeding | 90 (3) | 34 (2) |  | 48 (3) | 42 (3) |  | 67 (4) | 79 (3) |  | 0.001 | 0.13 |
| Transfusion | 36 (1) | 33 (2) |  | 43 (3) | 44 (3) |  | 88 (5) | 192 (7.5) |  | <0.001 | <0.001 |
| Death at 30 days | 38 (1) | 11 (0.5) |  | 53 (3) | 30 (2) |  | 190 (12) | 180 (7) |  | <0.001 | <0.001 |
| Death at one year | 90 (3) | 37 (2) |  | 94 (6) | 83 (6) |  | 359 (22) | 534 (21) |  | <0.001 | <0.001 |

Values are expressed as mean (± SD) or number (percentage)

MI, myocardial infarction, NSTEMI, non-ST-elevation myocardial infarction, STEMI, ST-elevation myocardial infarction

**Online Table 4.** Baseline characteristics according to survey

|  | **2005** | | | **2010** | | | **2015** | | | **P-value** | | |
| --- | --- | --- | --- | --- | --- | --- | --- | --- | --- | --- | --- | --- |
|  | **Low**  **(n=1185)** | **Interm**  **(n=853)** | **High**  **(n=1517)** | **Low**  **(n=1892)** | **Interm**  **(n=1029)** | **High**  **(n=1168)** | **Low**  **(n=2369)** | **Interm**  **(n=1226)** | **High**  **(n=1476)** | **2005** | **2010** | **2015** |
| Age (y) | 58.7±11.7 | 65.8±13.2 | 75.3±10.9 | 58.3±11.8 | 66.1±13.8 | 75.4±11.6 | 59.6±11.8 | 66.0±13.3 | 75.4±11.9 | <0.001 | <0.001 | <0.001 |
| Female | 244 (21) | 232 (27) | 636 (42) | 380 (20) | 317 (31) | 415 (36) | 486 (20.5) | 367 (30) | 535 (36) | <0.001 | <0.001 | <0.001 |
| BMI (Kg/m²) | 26.6±4.2 | 27.6±4.9 | 27.4±5.2 | 26.6±4.3 | 27.0±4.8 | 27.2±4.8 | 26.6±4.3 | 27.2±4.9 | 27.4±5.3 | <0.001 | <0.001 | <0.001 |
| **Risk factors** |  |  |  |  |  |  |  |  |  |  |  |  |
| Hypertension | 249 (21) | 563 (66) | 1304 (86) | 390 (21) | 758 (74) | 1038 (89) | 457 (19) | 931 (76) | 1326 (90) | <0.001 | <0.001 | <0.001 |
| Diabetes | 84 (7) | 296 (35) | 893 (59) | 45 (2) | 188 (18) | 580 (50) | 59 (2.5) | 261 (21) | 762 (52) | <0.001 | <0.001 | <0.001 |
| Hypercholesterolemia | 489 (41) | 424 (50) | 815 (54) | 679 (36) | 470 (46) | 622 (53) | 771 (32.5) | 588 (48) | 860 (58) | <0.001 | <0.001 | <0.001 |
| Current smoking | 407 (34) | 285 (33) | 344 (23) | 728 (38.5) | 426 (41) | 283 (24) | 853 (36) | 518 (42) | 390 (26) | <0.001 | <0.001 | <0.001 |
| **Medical history** |  |  |  |  |  |  |  |  |  |  |  |  |
| Prior MI | 110 (9) | 131 (15) | 409 (27) | 154 (8) | 160 (15.5) | 317 (27) | 234 (10) | 214 (17.5) | 485 (33) | <0.001 | <0.001 | <0.001 |
| Prior PCI | 104 (9) | 118 (14) | 283 (19) | 170 (9) | 161 (16) | 277 (24) | 239 (10) | 212 (17) | 477 (32) | <0.001 | <0.001 | <0.001 |
| Prior CABG | 8 (0.7) | 22 (3) | 173 (11) | 28 (1.5) | 62 (6) | 150 (13) | 7 (0.3) | 17 (1) | 184 (12.5) | <0.001 | <0.001 | <0.001 |
| History of heart failure | 4 (0.3) | 11 (1) | 195 13) | 3 (0.2) | 14 (1) | 152 (13) | 7 (0.3) | 30 (2) | 218 (15) | <0.001 | <0.001 | <0.001 |
| History of stroke | 13 (1) | 44 (5) | 241 (16) | 11 (0.6) | 38 (4) | 140 (12) | 32 (1) | 50 (4) | 216 (15) | <0.001 | <0.001 | <0.001 |
| Peripheral artery disease | 6 (0.5) | 25 (3) | 327 (22) | 11 (0.6) | 54 (5) | 252 (22) | 11 (0.5) | 48 (4) | 330 (22) | <0.001 | <0.001 | <0.001 |
| Chronic renal failure | 2 (0.2) | 18 (0.5) | 179 (5) | 5 (0.3) | 21 (2) | 145 (12) | 16 (0.7) | 26 (2) | 226 (15) | <0.001 | <0.001 | <0.001 |
| **Prior medications** | | | | | | | | | | | | |
| Aspirin | 148 (12.5) | 193 (23) | 566 (37) | 219 (12) | 219 (21) | 454 (39) | 286 (12) | 300 (24.5) | 700 (47) | <0.001 | <0.001 | <0.001 |
| Clopidogrel | 61 (5) | 83 (10) | 327 (22) | 95 (5) | 106 (10) | 299 (26) | 58 (2) | 83 (7) | 282 (19) | <0.001 | <0.001 | <0.001 |
| Betablockers | 135 (11) | 234 (27) | 525 (35) | 233 (12) | 271 (26) | 466 (40) | 315 (13) | 369 (30) | 659 (45) | <0.001 | <0.001 | <0.001 |
| Statins | 217 (18) | 239 (28) | 557 (37) | 339 (18) | 288 (28) | 501 (43) | 398 (17) | 397 (32) | 712 (48) | <0.001 | <0.001 | <0.001 |
| ACE-Inhibitors or ARB | 141 (12) | 306 (36) | 807 (53) | 273 (14) | 422 (41) | 650 (56) | 434 (18) | 500 (41) | 772 (52) | <0.001 | <0.001 | <0.001 |
| **Clinical presentation** |  |  |  |  |  |  |  |  |  |  |  |  |
| STEMI | 767 (65) | 478 (56) | 582 (38) | 1220 (64.5) | 581 (56.5) | 519 (44) | 1378 (58) | 611 (50) | 514 (35) | <0.001 | <0.001 | <0.001 |
| Killips class  I  II  III  IV | 1155 (97.5)  21 (2)  6 (0.5)  3 (0.3) | 750 (88)  69 (8)  23 (3)  11 (1) | 802 (53)  378 (25)  288 (19)  49 (3) | 1851 (98)  31 (2)  4 (0.2)  3 (0.2) | 912 (89)  80 (8)  23 (2)  8 (0.8) | 664 (57)  283 (24)  158 (13.5)  51 (4) | 2325 (98)  29 (1)  4 (0.2)  4 (0.2) | 1108 (90)  75 (6)  25 (2)  9 (0.7) | 977 (66)  261 (18)  183 (12)  42 (3) | <0.001 | <0.001 | <0.001 |
| LV function | 55±12 | 53±13 | 48±14 | 54±10 | 52±11 | 48±12 | 53±10 | 52±11 | 48.5±12 | <0.001 | <0.001 | <0.001 |
| GRACE score | 128.1±26.3 | 141.1±32.1 | 169.7±35.7 | 124.6±26.8 | 138.6±30.5 | 168.6±37.5 | 127.3±26.9 | 138.3±30.8 | 165.4±35.6 | <0.001 | <0.001 | <0.001 |
| SRI | 19.8±9.6 | 26.1±15.0 | 35.6±16.1 | 19.2±9.4 | 25.1±13.0 | 35.2±17.6 | 21.1±10.6 | 25.4±12.6 | 34.9±16.7 | <0.001 | <0.001 | <0.001 |
| CRUSADE | 18.9±11.1 | 26.4±12.7 | 44.5±14.2 | 20.4±11.2 | 27.8±13.0 | 43.6±14.4 | 17.4±10.9 | 24.3±12.9 | 40.3±15.4 | <0.001 | <0.001 | <0.001 |

Values are expressed as mean (± SD) or number (percentage)

ACE, angiotensin-converting enzyme; ARB, angiotensin receptor blockers; BMI, body mass index; CABG, coronary artery bypass grafting; CRP, C-reactive protein, LV, left ventricular; MI, myocardial infarction; PCI, percutaneous coronary intervention; SRI, simple risk index; STEMI, ST-elevation myocardial infarction.

**Online Table 5.** In-hospital management according to survey

|  | **2005** | | | **2010** | | | **2015** | | | **P-value** | | |
| --- | --- | --- | --- | --- | --- | --- | --- | --- | --- | --- | --- | --- |
|  | **Low**  **(n=1185)** | **Interm**  **(n=853)** | **High**  **(n=1517)** | **Low**  **(n=1892)** | **Interm**  **(n=1029)** | **High**  **(n=1168)** | **Low**  **(n=2369)** | **Interm**  **(n=1226)** | **High**  **(n=1476)** | **2005** | **2010** | **2015** |
| **Medications** |  |  |  |  |  |  |  |  |  |  |  |  |
| Aspirin | 1109(94) | 794(93) | 1328(87.5) | 1858(98) | 1001(97) | 1108(95) | 2159(91) | 1110(90.5) | 1300(88) | <0.001 | <0.001 | <0.001 |
| Clopidogrel | 1095(92) | 755(88.5) | 1196(79) | 1422(75) | 851(83) | 1013(87) | 374(16) | 303(25) | 618(42) | <0.001 | <0.001 | <0.001 |
| Ticagrelor | - | - | - | - | - | - | 1365(58) | 613(50) | 505(34) | - | - | <0.001 |
| Prasugrel | - | - | - | 673 (36) | 233(23) | 121(10) | 440(19) | 151(12) | 65(4) | - | <0.001 | <0.001 |
| GPIIbIIIa | 37(3) | 17(2) | 20(1) | 57(3) | 23(2) | 13(1) | 18(0.8) | 9(0.7) | 4(0.3) | <0.001 | <0.001 | <0.001 |
| UFH | 591(50) | 412(48) | 844(56) | 1143(60) | 432(42) | 577(49) | 1614(68) | 382(31) | 521(35) | 0.06 | <0.001 | 0.14 |
| LMWH | 805(68) | 537(63) | 782(51.5) | 1303(69) | 627(61) | 592(51) | 1115(47) | 577(47) | 506(34) | <0.001 | <0.001 | <0.001 |
| Bivalirudine | - | - | - | 65(3) | 38(4) | 29(2.5) | 87(4) | 36(3) | 20(1) | - | 0.22 | <0.001 |
| Fondaparinux | - | - | - | 316(17) | 176(17) | 163(14) | 563(24) | 290(24) | 426(29) | - | 0.07 | 0.001 |
| Statins | 988(83) | 663(78) | 1004(66) | 1517(80) | 795(77) | 811(69) | 1981(84) | 971(79) | 1090(74) | <0.001 | <0.001 | <0.001 |
| Beta-blockers | 941(79) | 643(75) | 894(59) | 1622(86) | 835(81) | 851(73) | 1742(73.5) | 883(72) | 979(66) | <0.001 | <0.001 | <0.001 |
| ACE-inhibitors or ARB | 579(49) | 464(54) | 867(57) | 1191(63) | 702(68) | 725(62) | 1425(60) | 792(65) | 865(59) | <0.001 | 0.005 | 0.004 |
| **Procedures** |  |  |  |  |  |  |  |  |  |  |  |  |
| CAG | 1150(97) | 773(91) | 1087(72) | 1874(99) | 995(67) | 1004(86) | 2357(99.5) | 1207(98.5) | 1353(92) | <0.001 | <0.001 | <0.001 |
| PCI | 912 (77) | 597 (70) | 754 (50) | 1595 (84) | 841 (82) | 770 (66) | 1977 (83.5) | 993 (81) | 980 (66) | <0.001 | <0.001 | <0.001 |
| Reperfusion therapy in STEMI patients  Primary PCI  Fibrinolysis  Medical therapy | 288 (37.5)  271 (35)  200 (27) | 187 (39)  132 (28)  158 (33) | 186 (32)  98 (17)  298 (51) | 821 (67)  199 (16)  200 (16) | 387 (67)  85 (15)  109 (19) | 299 (58)  53 (10)  167 (32) | 962 (70)  98 (7)  318 (23) | 423 (69)  27 (4)  161 (26) | 310 (60)  14 (3)  190 (37) | <0.001 | <0.001 | <0.001 |
| CAG <24hours in NSTEMI patients | 172 (43) | 130 (39) | 204 (32) | 343 (52) | 187 (44) | 176 (33) | 563 (57) | 323 (54) | 360 (42) | <0.001 | <0.001 | <0.001 |

Values are expressed as mean (± SD) or number (percentage)

ACE, angiotensin-converting enzyme; ARB, angiotensin receptor blockers; CAG, coronary angiography; LMWH, low-molecular-weight heparin; NSTEMI, Non- ST-elevation myocardial infarction; PCI, percutaneous coronary intervention; STEMI, ST-elevation myocardial infarction; UFH, unfractionated heparin.

**Online Table 6.** In-hospital complications and clinical outcomes according to acute myocardial infarction

|  | **2005** | | | **2010** | | | **2015** | | | **P-value** | | |
| --- | --- | --- | --- | --- | --- | --- | --- | --- | --- | --- | --- | --- |
|  | **Low**  **(n=1185)** | **Interm**  **(n=853)** | **High**  **(n=1517)** | **Low**  **(n=1892)** | **Interm**  **(n=1029)** | **High**  **(n=1168)** | **Low**  **(n=2369)** | **Interm**  **(n=1226)** | **High**  **(n=1476)** | **2005** | **2010** | **2015** |
| Re-MI | 11(0.9) | 14(2) | 47(3) | 18(1) | 9(0.9) | 14(1) | 3(0.1) | 3(0.2) | 11(0.7) | <0.001 | 0.72 | 0.004 |
| Intrastent thrombosis | - | - | - | 8(0.4) | 5(0.5) | 8(0.7) | 17(0.7) | 7(0.6) | 8(0.5) | - | 0.61 | 0.76 |
| Atrial fibrillation | 38(3) | 28(3) | 132(9) | 69(4) | 68(7) | 162(14) | 67(3) | 71(6) | 135(9) | <0.001 | <0.001 | <0.001 |
| Ventricular fibrillation | 24(2) | 16(2) | 29(2) | 35(2) | 19(2) | 23(2) | 51(2) | 19(1.5) | 29(2) | 0.97 | 0.97 | 0.46 |
| Stroke | 7(0.6) | 5(0.6) | 21(1) | 5(0.3) | 4(0.4) | 6(0.5) | 4(0.2) | 7(0.6) | 16(1) | 0.05 | 0.54 | 0.001 |
| Major bleeding | 11(0.9) | 12(1) | 55(4) | 32(2) | 19(2) | 34(3) | 34(1) | 22(2) | 40(3) | <0.001 | 0.03 | 0.003 |
| Minor bleeding | 8(0.7) | 8(0.9) | 18(1) | 44(2) | 26(2.5) | 42(4) | 72(3) | 56(5) | 86(6) | 0.40 | 0.04 | 0.05 |
| Transfusion | 16(1) | 24(3) | 108(7) | 30(2) | 31(3) | 72(6) | 23(1) | 32(3) | 100(7) | <0.001 | <0.001 | <0.001 |
| Death at 30 days | 21(2) | 36(4) | 173(11) | 11(0.6) | 19(2) | 84(7) | 17(0.7) | 28(2) | 113(8) | <0.001 | <0.001 | <0.001 |
| Death at one year | 43(4) | 67(8) | 390(26) | 40(2) | 55(5) | 237(20) | 44(2) | 55(4.5) | 266(18) | <0.001 | <0.001 | <0.001 |

Values are expressed as mean (± SD) or number (percentage)

MI, myocardial infarction

**Online Figure 1.** TRS2P categories between 2005 to 2015

**Online Figure 2.** Distribution of the 9 variables according to TRS-2P categories

CABG, coronary artery bypass graft; CHF, congestive heart failure; DM, diabetes mellitus; eGFR, estimated glomerular filtration rate; HTN, hypertension; PAD, peripherical artery disease
